# Men's Perceptions of Non‐Cancer Related Lymphoedema: A Qualitative Diary Study

**DOI:** 10.1111/nhs.70201

**Published:** 2025-08-05

**Authors:** Garry Cooper, Nicola Gale, Manbinder Sidhu, Kerry Allen

**Affiliations:** ^1^ University of Birmingham UK

**Keywords:** lymphoedema, masculinity, men, narrative, perceptions, vulnerability

## Abstract

Lymphoedema is a chronic condition caused by lymphatic dysfunction, resulting in persistent swelling. Although men are significantly affected, their experiences, particularly regarding masculine identity, remain underexplored. This UK‐based study examined how men live with non‐cancer‐related lymphoedema, drawing on hegemonic masculinity and the Health, Illness, Men, and Masculinities (HIMM) framework. A qualitative narrative inquiry was conducted with 12 men who completed solicited diaries over 2 weeks. Thematic and structural analyses identified one overarching theme—vulnerability—and two subthemes: lymphoedema as a constant reminder of disruption, and routine as a means of control and masculine continuity. Participants described concealment, emotional strain, and social withdrawal, especially in response to stigma and misunderstanding in public and clinical settings. Many developed routines to regain autonomy, preserve identity, and express competence. Some upheld traditional masculine traits such as stoicism, while others redefined masculinity through reflection, disclosure, or relational support. These findings show how men negotiate identity in the context of chronic illness and adapt to long‐term self‐management. The study highlights the need for gender‐sensitive care that recognizes how masculinities shape the lived experience of lymphoedema.

**Key Points**

- Men with lymphoedema frequently encounter vulnerability, often triggered by a lack of awareness among healthcare professionals and the public. Participants described negative encounters, including misinterpretation of symptoms in public or clinical settings, that led to frustration, concealment, and social withdrawal.
- The condition functioned as a constant reminder of change, disrupting daily activities, relationships, and masculine identity. Physical limitations and emotional strain challenged men's sense of competence, while efforts to maintain normalcy revealed tensions between independence and reliance on others.
- Routine emerged as a key strategy for regaining control. Daily self‐management practices—such as compression therapy and symptom monitoring—helped men structure their lives and maintain a sense of agency. These routines supported emotional resilience and provided continuity with pre‐illness roles.

## 1 Introduction

Lymphoedema is a chronic, incurable condition that causes swelling due to lymphatic failure and is associated with infection, hospitalization, reduced mobility, and diminished quality of life (Fletcher et al. 2024; Quéré et al. 2019). Despite men comprising 15%–22% of UK lymphoedema service caseloads, with a recorded prevalence of 2.48 per 1000 compared to 5.37 per 1000 in women (Cooper and Bagnall 2016; Moffatt et al. 2017, 2019), their experiences remain under‐researched and under‐recognized. This underrepresentation mirrors a broader pattern of epistemic and clinical neglect of lymphoedema in men as a chronic condition (Cooper‐Stanton et al. 2022; Moffatt et al. 2019).

Men's health often remains marginalized and neglected in both policy and practice (Brine 2024; UK Parliament 2013a, 2023, 2024; WHO 2018; Womens and Equalities Committee 2018). In the UK, men experience shorter life expectancy 4 years shorter than women, but higher rates of non‐communicable disease, social isolation, and lower levels of help‐seeking (Brine 2024; GAOMH 2020, 2021; McKenzie et al. 2018; ONS 2022a, 2022b, 2022c; Robertson and Baker 2017; WHO 2018). These intersecting patterns may contribute to the concealment, underdiagnosis, and psychosocial burden of lymphoedema among men (Cooper‐Stanton et al. 2022; ILF 2006; Fletcher et al. 2024).

While most research on lymphoedema focuses on women and cancer‐related causes, few studies explore how men perceive and adapt to non‐cancer‐related lymphoedema (Cooper‐Stanton et al. 2022). Given the gendered nature of chronic illness, researchers must consider how men negotiate masculine identity, stigma, and care. This study uses solicited diaries, a qualitative method that allows participants to document their lived experience over time with minimal researcher interference (Meth 2017; Milligan et al. 2005). This method enables exploration of gendered narratives while minimizing “gendered performance” bias (Broom et al. 2009, p. 52).

Guided by hegemonic masculinity and the Health, Illness, Men, and Masculinities (HIMM) framework, we examine how men perceive and respond to living with lymphoedema in daily life (Connell and Messerschmidt 2005; Evans et al. 2011). These frameworks offer insight into how masculinity is performed, disrupted, and reconfigured in response to chronic illness. Our study addresses the following research question: How do men perceive their non‐cancer‐related lymphoedema within their daily lives?

### 1.1 Background

Lymphoedema can arise from primary causes, such as genetic abnormalities, or secondary factors including trauma or surgical intervention, and it frequently coexists with long‐term conditions such as diabetes, heart failure, and neurological disorders (Cooper and Bagnall 2016; ILF 2006; Keeley et al. 2019; Rankin 2016). These comorbidities are more prevalent among men in the UK (Bellanca et al. 2023; ONS 2024), yet researchers continue to overlook gendered analyses in the lymphoedema literature. Men's experiences of chronic illness are often shaped by dominant masculine norms that emphasize stoicism, physical strength, and autonomy. When chronic conditions limit mobility, independence, or social participation, they may disrupt masculine identity and exacerbate feelings of isolation (Courtenay 2009a; Flurey et al. 2018). This is particularly relevant given lymphoedema's need for sustained self‐management and low public but also healthcare professional awareness (Cooper‐Stanton et al. 2022; Scerri et al. 2024).

While compression therapy and psychosocial support can reduce complications and enhance quality of life, outcomes often depend on sustained adherence, access to care, and patient engagement (Cooper‐Stanton et al. 2022). However, men face distinct barriers, including limited awareness, stigma, and the absence of gender‐sensitive services, which remain underexplored in both research and policy (Nairn et al. 2019; Cooper‐Stanton et al. 2022). Our study responds to these gaps and contributes to the growing call for gender‐informed approaches to long‐term condition management by examining how men experience and navigate lymphoedema in relation to masculine identity (Evans et al. 2011; Galdas and Cheater 2000). Situating lymphoedema within the broader discourse on men and chronic illness strengthens the conceptual grounding for our analysis, which is outlined in the following section.

### 1.2 Conceptual Frameworks

Our study draws on the conceptual frameworks of hegemonic masculinity and the Health, Illness, Men, and Masculinities (HIMM) framework to explore how men with lymphoedema navigate shifts in masculine identity. These frameworks provide structured lenses to examine the socially constructed and performative nature of masculinity and how chronic illness may disrupt or reshape men's self‐perceptions (Connell and Messerschmidt 2005; Evans et al. 2011). Hegemonic masculinity refers to a dominant cultural ideal of manhood, characterized by traits such as strength, independence, and assertiveness, often enacted through domains like sport, work, and fatherhood (Bosson et al. 2021; Galdas and Cheater 2000; Connell and Messerschmidt 2005; Messerschmidt 2019). Masculinity, described as a “pattern of practice,” is performed for both self and others and is not static or universal. It shifts across global, regional, and local contexts in response to broader political and cultural changes (Connell and Messerschmidt 2005, p. 832; Galdas and Cheater 2000).

When men cannot perform masculinity in expected ways, due to physical limitations from chronic illness, their social status may be destabilized, and others may perceive them as subordinate (Courtenay 2009b; Valved et al. 2021). However, they also resist, reject, or reinterpret hegemonic ideals, particularly when those ideals feel restrictive or unattainable. Under such conditions, alternative expressions of masculinity, including inclusive or subordinate forms, may emerge (Galdas and Cheater 2000; Messerschmidt 2019).

The HIMM framework complements this perspective by examining how masculinity shifts across the life course in relation to health and illness (Evans et al. 2011; Messerschmidt 2019). It positions masculinity as a social determinant of health, shaped by intersecting factors such as age, sexuality, and chronic illness. Together, these frameworks offer a gender‐sensitive lens through which to understand men's experiences of lymphoedema, highlighting the psychosocial complexity of adapting to the condition. Guided by these frameworks, our study addresses the following research question:

1. How do men perceive their non‐cancer‐related lymphoedema within their daily lives?

## 2 Methods

### 2.1 Design

We conducted a qualitative narrative inquiry using solicited diaries to explore participants' lived experience and perceptions of lymphoedema over time (Clandinin 2012, Milligan et al. 2005). Solicited diaries enabled participants to shape the depth, timing, and content of their entries, thereby reducing researcher intrusion and enhancing authenticity (Broom et al. 2009). This autonomy allowed men to reflect on their experiences in their own terms, offering rich insights into masculinity, illness, and identity. While this approach supports participant‐led narrative construction, a recognized limitation is the absence of follow‐up questioning to explore emergent meaning in more depth (Pino Gavidia and Adu 2022).

### 2.2 Access, Recruitment, and Sampling

We purposively recruited participants from a previous UK and Ireland study on men with non‐cancer‐related lymphoedema. Over a 12‐month period (September 2021 to August 2022), we promoted recruitment through national organizations, healthcare professionals, and social media platforms, including X (formerly Twitter). While debates continue around sample size in qualitative research, narrative inquiry typically does not rely on pre‐determined numbers (Braun et al. 2015; Tran et al. 2017). Similarly, views on data saturation vary; we determined sufficiency based on the depth and richness of the data collected (Caine et al. 2013). Our final sample of 12 men allowed for meaningful exploration of all interpretive avenues.

Participants were eligible if they met the following inclusion criteria: (1) aged 18 years or older, (2) self‐identified as a man, (3) had a diagnosis of primary or secondary lymphoedema (non‐cancer‐related), and (4) resided in the United Kingdom (all four nations) or the Republic of Ireland. Each participant was screened against these criteria before providing informed consent. Diaries were distributed by email or post, accompanied by guidance and a submission date. Participants also provided demographic details, including age, education, ethnicity, occupation, location, and diagnosis duration, to consider socioeconomic and intersectional factors in the analysis (Table 1). Participants were aged 34–77, with most between 50 and 59 years; further details are shown in Table 1.

**TABLE 1:** Participant characteristics (*N* = 12).

Characteristic	Category	*N*	%	Participants (Pseudonyms and age)
Age	30–39	3	25.00%	Luke (34), Alfie (37), Ryan (39)
	40–49	1	8.30%	Harry (41)
	50–59	6	50.00%	Mike (54), Will (55), George (56), Henry (56), Chris (50), Jake (54)
	70–80	2	16.70%	Arthur (70), Ben (71)
	Mean age (years)*		52.1	
Ethnicity	White–British	10	83.30%	
	White–Irish	1	8.30%	
	White–Scottish	1	8.30%	
Highest qualification	GCSE	1	8.30%	
	Apprenticeship	1	8.30%	
	A‐Level	1	8.30%	
	Degree	7	58.30%	
	Masters	2	16.70%	
Diagnosis type	Primary Lymphoedema	10	83.30%	
	Secondary Lymphoedema	2	16.70%	
Diagnosis duration	0–9 years	5	41.70%	
	10–19 years	4	33.30%	
	20–42 years	3	25.00%	
	Mean duration (years)*		14.5	

* Percentages based on *N* = 12. Mean values calculated using midpoints of age and diagnosis duration ranges.

### 2.3 Data Collection

Participants completed their two‐week diaries at home between October 2021 and May 2022. We followed Meth (2017, p. 105) “Steps to Using Solicited Diaries” and template to ensure a structured approach and to encouraging participation (Kenten 2010; Meth 2017). The structure included when (date of event), what (situation/event details), how (feelings related to the event), whom (did they speak to anyone about the event), and other (to add further details if needed). Participants had flexibility in how they completed, providing entries daily, on alternate days, or weekly. This flexible approach allowed participants to share their narratives in depth and their own terms (Milligan et al. 2005; Kenten 2010; Meth 2017; Milligan et al. 2005). We transferred the completed data into and managed it in Microsoft Excel for analysis.

### 2.4 Ethics

The University of Birmingham Ethics Committee (ERN_20–1729; August 18, 2021) approved the study in accordance with the Declaration of Helsinki (World Medical Association 2024). All participants provided informed consent before taking part. We anonymized data by removing identifiable information and using pseudonyms, as illustrated in Table 1. In line with the Data Protection Act (UK Government 2018) and EU General Data Protection Regulation (GDPR), we stored all data securely on a password‐encrypted computer. Participants did not receive financial incentives for participation.

### 2.5 Data Analysis

We used a two‐stage analytic approach grounded in narrative inquiry: structural analysis (Labov 1972) followed by narrative thematic analysis (Riessman 2008). We examined how participants told their stories using Labov's six narrative elements: abstract (summary), orientation (who/when/where), complicating action (main events), evaluation (personal commentary), resolution (outcome), and coda (return to the present) (Hunter 2011; Labov 1972). This helped us understand how men framed and structured their experiences of lymphoedema. We then conducted narrative thematic analysis to explore what participants said about masculinity, identity, and vulnerability (Riessman 2008). We developed codes inductively through repeated readings of the diaries and refined them through team discussions and feedback from participants. We paid particular attention to language, metaphor, and self‐positioning to ensure that the themes reflected both individual experiences and shared social meanings. This dual approach enabled us to explore how men positioned themselves in relation to dominant cultural narratives of masculinity (Hunter 2011) and to interpret these in light of the conceptual frameworks described earlier (Connell and Messerschmidt 2005; Courtenay 2009a).

Reflexivity played a central role in our analytic process, particularly given our professional backgrounds, assumptions, and personal characteristics. These factors inevitably influenced how we approached the study. The lead researcher is a cisgender man and a nurse specialist with experience managing lymphoedema. His background provided valuable clinical insights but also introduced potential biases, especially around professional assumptions related to men's health, masculinity, and chronic illness management. To manage these influences transparently, a reflexive diary was kept throughout the study, recording insights, assumptions, reactions, and decisions.

Our research team held regular meetings to critically reflect on how personal and professional experiences, assumptions, and presuppositions might shape the research questions, methodological choices, data collection, analysis, and interpretation. During these meetings, we specifically considered how the research team's identity, clinical experience, and relationships with participants could influence data interpretation and the framing of our findings.

We adopted Mauthner and Doucet (1998, 2003) relational approach to reflexivity, recognizing that we co‐constructed the meanings in the data through our interactions with participants and among our research team. Throughout the analysis, we shared provisional themes and interpretations with co‐authors and participants, actively seeking critical feedback to enhance interpretive rigor and to ensure our findings authentically reflected participants' experiences (Parahoo 2014; Riessman 2008). This collaborative reflexive process strengthened transparency, methodological rigor, and the transferability of our findings to similar contexts.

## 3 Results

The analysis of the solicited diaries led to the identification of one overarching theme, vulnerability, with two subthemes: (1) lymphoedema as a constant reminder of change and (2) routine as a source of control and masculine affirmation. These findings are summarized in Table 2, which provides brief descriptors and illustrative participant quotes to support the thematic structure. The table also serves as a visual guide to the results presented in this section and should be read alongside the narrative accounts below.

**TABLE 2:** Study's findings organized into overarching themes identified through structural and narrative thematic analysis.

Main theme	Subthemes
Vulnerability‑“limited understanding or interest*”*‑Participants described a sense of vulnerability linked to poor public and clinical awareness of lymphoedema, contributing to stigma, misrecognition, and concealment.	A constant reminder–“affects you day to day”–Lymphoedema was perceived as a daily disruption to mobility, parenting, and self‐image. It reminded participants of their differences, contributing to emotional strain and self‐consciousness.
Illustrative Quotes: “This was the third vascular consultant I have seen… They have a very limited understanding or interest in the condition.” (Alfie) “Went Christmas shopping… I heard a couple behind me say I must be drunk.” (Henry)	Illustrative Quotes: “This condition affects you day to day.” (Jake) “My son said, “My dad cannot race—he is got a rubbish leg.” I was embarrassed.” (Mike)
	Solace in Routine–“Early morning routine as always”–Participants developed personal routines to regain control, maintain independence, and reassert their masculine identity through self‐discipline and expertise.
	Illustrative Quotes: “Early morning routine as always. Aware of leg and check swelling.” (Ben) “I like to regard myself as an expert patient.” (Ben)

### 3.1 Main Theme: Vulnerability‑“Limited Understanding or Interest”: Experiences of Stigma, Invisibility, and Concealment

Many participants described a persistent sense of vulnerability, often triggered by public and professional misunderstanding of lymphoedema. This vulnerability manifested in frustration, isolation, and a reluctance to disclose their condition. These feelings shaped how participants navigated interactions and made decisions about when and how to speak about their experiences. Several men described dismissive or disheartening interactions with healthcare professionals, which left them feeling frustrated and unsupported. Alfie recounted one such experience: “This was the third vascular consultant I have seen… They have a very limited understanding or interest in the condition. This consultant looked at both my legs and said they looked fine, just continue wearing my compression stockings. Altogether, the consultation was about 3 min in duration.”

This brief and impersonal exchange reflects a broader pattern in which men perceived their condition as being minimized or overlooked. Such encounters not only discouraged help‐seeking but also reinforced feelings of invisibility and marginalization within healthcare.

The sense of being misunderstood was not limited to clinical contexts. Henry described a public encounter that left him feeling judged and embarrassed: “Went Christmas shopping, but due to the lymphoedema, I cannot walk in a straight line. I heard a couple behind me say I must be drunk… I could not wait to get back home.” Later that same day, Henry was stopped by the police, who interpreted his mobility as a sign of intoxication. Even after explaining his condition, he received neither acknowledgement nor apology. These incidents highlight the social stigma that can accompany visible symptoms and underscore the emotional toll of being misread in public settings.

These experiences highlight how men's sense of control, competence, and social standing could be undermined when their bodies are perceived as different or impaired. Visible symptoms often trigger negative responses or assumptions from others, reinforcing feelings of marginalization and the need for self‐protection. This sense of vulnerability extended into both professional and personal contexts. For example, Alfie described concealing his condition during a workplace well‐being meeting: “One of the team members is trying to encourage more physical activity, like cycling and running, in the wider team. I am still uncomfortable sharing any more details about my condition, in part because I feel people are unfamiliar and do not understand it, therefore cannot relate to it.” Here, silence served as a protective strategy to avoid judgment or misrecognition in a setting where physical capability was implicitly valued.

The same tension between openness and concealment shaped how men engaged socially. Alfie also reflected on his reluctance to share a national news article about lymphoedema with friends: “Despite wanting to, I chose not to share it because I did not want to be identified as a sufferer.” This decision underscores how the desire to raise awareness was complicated by fears of stigma and exposure. For some, staying silent was a means of preserving social identity, even at the expense of advocacy or support. Not all men choose concealment. Luke described becoming involved in advocacy: “They want me to contribute to their website and magazine… there needs to be more positivity about lymphoedema.” Luke's involvement suggested a shift in identity through openness and contribution, reflecting how some men redefined themselves in response to living with a long‐term condition.

Vulnerability was thus experienced in social, clinical, and professional contexts, shaped by external perceptions and men's strategic responses. For some, this prompted withdrawal; for others, it fostered redefinition and advocacy. While vulnerability often emerged in response to misunderstanding and concealment, men also described how lymphoedema disrupted their everyday routines, physical capabilities, and relationships. The condition acted as a constant presence, reminding them of change, loss, and the need to adapt. These daily reminders, explored in the next theme, further shaped how participants negotiated their identities and adjusted to living with a long‐term condition.

### 3.2 Sub‐Theme 1: A Constant Reminder—“Affects You Day to Day”: Disruption to Daily Life, Mobility, and Identity

Many participants described lymphoedema as a persistent presence in their daily lives‐something that shaped how they moved, felt, and related to others. Jake explained: “Waking up for work again, my leg is feeling fat… this condition affects you day to day.” His account illustrates the discomfort and constant awareness that shaped even simple daily activities, reinforcing the ongoing impact of the condition. These feelings often contributed to frustration and fatigue, which influenced how men approached tasks that once felt effortless.

Similarly, Ryan noted: “Every morning, I wake up with something different… It is been such a fight trying to find out what is happening.” His experience echoed a broader theme of unpredictability, which undermined participants' sense of control and stability. For many, mornings brought renewed uncertainty about their body's function, prompting worry and adaptation.

Routine public interactions also became a source of discomfort and vigilance. Mike shared:

“Usually, I do not like to and cannot properly readjust the compression hosiery in a public space. I have to find a public restroom in a café or shop.” This quote reveals how managing the condition in public settings could be awkward and physically limiting. Participants often described these adjustments as inconvenient and isolating, which reinforced their sense of difference and diminished spontaneity. Despite this, men often sought to preserve routines that reflected normalcy and masculine identity. Harry described an outing that combined physical activity with ongoing symptom monitoring: “I have had a long walk today. After climbing rocks along the coast, the skin on my lower left leg looks incredibly dry, even after a shower. A common theme with this condition is frustration and remembering what it was like to not have to worry about these issues.” This account illustrates how some men continued to engage in activities linked to independence and competence, even while managing discomfort. These moments often involved both determination and a subtle mourning for their former physical freedom.

The emotional impact of lymphoedema was particularly evident in family life. Mike recounted a moment with his son: “In a play area with my son, I overheard him discussing my leg with a child he was playing with at the time. My son said, “My dad cannot race, he's got a rubbish leg.” I was embarrassed and annoyed with myself.” This interaction highlights how shifting physical ability affected men's roles as parents. Feelings of embarrassment, frustration, and inadequacy emerged when participants were reminded, especially by children, of what they could no longer do.

Dependence on others further challenged men's sense of autonomy. Jake explained: “I had two choices: ask for help or wait for my wife at the store, or wait until my wife was free to help. I opted for the latter. I felt completely useless and dependent on other people to do simple tasks.” These reflections reveal the emotional cost of relying on others for basic activities. Avoiding help was, for some, a way to preserve dignity—even when it led to further limitations. Some diary entries captured reflective moments triggered by milestones. Alfie wrote: “My birthday has caused me to reflect on how difficult the last few years have been. I worry about my lymphoedema getting worse.” Here, a routine event prompted deeper introspection about the condition's trajectory and the uncertainty it introduced. These moments added emotional weight to the day‐to‐day reality of living with lymphoedema.

For others, intimate relationships offered space for emotional acceptance. Luke shared:

“I wanted to show I had accepted my condition… my boyfriend knows the most about how this condition affects me.” Luke's account demonstrates how vulnerability could be expressed within trusted relationships. Acceptance, for some, was not only internal but relational, facilitated by partners who understood the condition's impact.

These varied accounts of disruption and reflection underscore the emotional and physical persistence of lymphoedema in everyday life. While the condition was a constant reminder of limitation, it also prompted efforts to reclaim stability, often through structured routines.

### 3.3 Sub‐Theme 2: Routine—“Early Morning Routine as Always: Regaining Control, Structure, and Masculine Continuity

Routine emerged as a vital strategy through which men managed the daily impact of lymphoedema. These routines were not only practical responses to physical symptoms but also shaped how participants reasserted stability, control, and identity in the face of ongoing disruption. The repetitive nature of self‐care tasks became a means to create structure and reclaim a sense of agency. Ben wrote: “Early morning routine as always. Aware of leg and check swelling.” Though brief, this entry reflects the embedded nature of daily self‐monitoring in participants' lives. For Ben and others, checking their condition each morning became part of their normal routine—both an act of vigilance and a means of establishing control.

These habits helped men navigate a condition marked by uncertainty and, in some cases, reassured them that they were doing everything possible to prevent deterioration.

Luke described the ongoing adjustments required throughout his day: “Having to adjust my compression stockings throughout the day. I feel frustrated, but I can at least be active throughout the day. I do not usually speak to anybody; it is a common part of life with this condition.” Luke's comment illustrates how participants balanced frustration with functionality. Although these adjustments were physically and emotionally taxing, they enabled men to remain active and preserve a sense of normality. The decision not to discuss these routines with others also suggests that self‐management became both a private task and a measure of personal resilience.

Over time, some men developed a strong sense of expertise in managing their condition. Ben reflected: “Once I overcame the shock that I had the condition for life. My concern has been to manage it well and avoid cellulitis. My fear is that it will get harder to manage. I like to regard myself as an expert patient.” This self‐identification as an “expert patient” revealed how routine could serve as a source of pride and competence. For Ben, the discipline and knowledge developed over time allowed him to feel in control of his health. The role of expert also offered an alternative identity to that of “patient,” positioning him as proactive rather than passive. Although routines varied in complexity and focus, they served as anchors in participants' lives. From early morning checks to compression garment care, these repetitive actions provided a sense of order. They allowed men to feel capable and structured, even as they faced physical limitations. For many, routine was not merely a health necessity, but a stabilizing force that helped to preserve continuity with who they were before diagnosis.

Together, these themes reveal the everyday challenges and adaptive strategies that shaped men's experiences of lymphoedema. Vulnerability, disruption, and routine were not experienced in isolation but interacted in complex ways, influencing how participants managed their condition and navigated masculine identity. These findings provide a foundation for interpreting how men negotiated meaning, care, and selfhood—issues we now explore through the lens of theory in the discussion that follows.

## 4 Discussion

This study explores how men experience non‐cancer‐related lymphoedema and how they manage its impact on daily life. At the time of writing, no published research has combined men's experience of this condition with conceptual frameworks, such as hegemonic masculinity and the HIMM framework (Connell and Messerschmidt 2005; Evans et al. 2011). Through solicited diary entries, we identified one overarching theme and two sub‐themes, which are now interpreted in relation to wider literature and relevant theoretical perspectives.

### 4.1 Vulnerability—“Limited Understanding or Interest”: Disruptions to Masculinity

Men's experiences of vulnerability were not confined to traditional “life event's” described in the HIMM framework (Evans et al. 2011). Instead, vulnerability was shaped by emotional suppression, misrecognition, and the stigma surrounding lymphoedema. Limited awareness among healthcare professionals and the wider public contributed to delays in diagnosis and poor support patterns consistent with existing evidence (Moffatt et al. 2003; Schulze et al. 2018; UK Parliament 2013b). This dynamic reinforces Hamilton and Thomas's (2016, p. 4) observation that lymphoedema is both “highly visible” and “largely invisible” in society.

Although hegemonic masculinity acknowledges marginalization when men cannot meet idealized norms (Connell and Messerschmidt 2005), it does not fully capture the personal disruption reported here. Bury's (1982) concept of “biographical disruption” provides a useful lens, revealing how chronic illness fractured participants' sense of identity. Many initially tried to maintain pre‐illness masculine ideals but later renegotiated these values, a shift echoed in other chronic illness studies (Charmaz 1995; Flurey et al. 2018).

Participants used concealment as a protective strategy, often motivated by fear of appearing weak or being misunderstood (Cooper‐Stanton et al. 2022; Flurey et al. 2018). This reflects Goffman's (1963) and Stangl et al.'s (2019) arguments that stigma is linked to traits seen as socially discrediting. Men's interactions with healthcare and law enforcement, where their condition was doubted or misread, exemplify the external reinforcement of this perceived stigma (Hackett et al. 2020). These findings align with wider debates about a ‘crisis of masculinity’ in which traditional norms are increasingly destabilized by changing roles and expectations (Kimmel 2017a, 2017b). While this was not the primary focus of the study, participants' behaviors, such as concealment, control, or routine, can be understood as responses to dominant masculine scripts. Such acts are best viewed as adaptive strategies shaped by social context, rather than personal failings (Jewkes et al. 2015; Latiff 2022).

### 4.2 A Constant Reminder—“Affects You Day to Day”: Embodied Change and Relational Disruption

Lymphoedema functioned as a constant physical and psychological presence in men's lives. This aligns with existing literature on chronic illness and “treatment burden”, which describes the cumulative impact of managing ongoing symptoms, appointments, and self‐care (Demain et al. 2015; ILF 2006). Both the HIMM framework and hegemonic masculinity highlight how illness can limit men's ability to perform culturally expected roles (Connell and Messerschmidt 2005; Evans et al. 2011). In this study, participants experienced disruption across work, parenting, and leisure domains central to masculine self‐perception.

These disruptions extended beyond physical ability. Men reflected on biographical and relational changes, describing uncertainty about the future and shifts in how they saw themselves. Such reflections support Bury's (1982) concept of “biographical disruption” and echo Charmaz's (1995) findings on the “loss of self” in chronic illness. Although some participants attempted to maintain masculine roles, others gradually adapted their values and behaviors, illustrating the flexibility of masculine identity in the face of illness (Flurey et al. 2018).

Participants who avoided certain activities also risked becoming more isolated. Men who had previously taken pride in physical competence or independence sometimes chose to withdraw rather than explain their condition, which reinforced feelings of social exclusion. These behaviors are consistent with broader findings in the literature on long‐term conditions and men's social participation (Demain et al. 2015). Help‐seeking, often culturally framed as feminine, was reframed by some participants as a pragmatic or courageous act. When such help was accepted—particularly from partners—it became a shared experience rather than a loss of autonomy (Arnell 2014). This finding supports the idea that masculinity can be renegotiated in illness, blending resilience with relational openness. Acts of disclosure and reliance, in this context, became tools for adapting identity rather than evidence of failure. Such negotiations reflect the dynamic and performative nature of masculinity (Wilde et al. 2020, p. 314).

### 4.3 Routine—“Early Morning Routine as Always”: Reclaiming Control and Masculine Capital

While lymphoedema disrupted daily life, many participants developed structured routines as a means of restoring order and autonomy. These routines—often involving compression therapy, symptom monitoring, and self‐management—helped men regain a sense of control over their bodies and day‐to‐day functioning. The routines provided predictability, counterbalancing the physical unpredictability of the condition. Participants described routine not only as a coping mechanism but also as a means of performing responsibility and resilience. These practices supported identity preservation by enabling men to maintain their usual roles, such as worker, partner, or parent. In this way, routines became a form of “masculine continuity,” allowing men to uphold values such as discipline, self‐reliance, and competence, even in the face of limitation. This mirrors findings in other chronic illness research, where control is maintained through ritualized behaviors (Charmaz 1995; Flurey et al. 2018).

The concept of “expert patient” was introduced by one participant as a way of reframing dependency. Taking responsibility for treatment, preventing complications, and mastering symptom management helped men feel informed and capable. As Flurey et al. (2018) and Evans et al. (2011) note, such adaptations suggest that masculine identity is not lost in illness but redefined through new forms of agency. These behaviors challenge a simplistic view of masculinity as fixed or incompatible with vulnerability. Instead, they support a more relational and flexible model, where care, discipline, and responsibility are integrated into the performance of masculinity. Participants' routines reflected not a retreat from identity, but an evolution of it, rooted in lived experience, embodiment, and context.

This study highlights how men with non‐cancer‐related lymphoedema navigated vulnerability, disruption, and identity through daily life. Participants responded to stigma and misunderstanding with strategies such as concealment, reflection, and routine. Self‐management practices provided control and continuity, allowing men to maintain valued aspects of masculinity while adapting to physical and social change. These findings support a dynamic view of masculinity in illness and underline the need for gender‐sensitive, relational approaches to long‐term condition care. We consider the implications for practice in the concluding sections that follow.

### 4.4 Strengths and Limitations

This study offers an in‐depth exploration of the daily experiences of men diagnosed with non‐cancer‐related lymphoedema. By applying theoretical frameworks not previously used in this context, it adds conceptual depth to the literature on men's experiences of lymphoedema. The sample—predominantly White men over the age of 45—reflects national and international trends in developed Western countries (Keeley et al. 2019; Moffatt et al. 2019). Including participants across a range of ages supports a life course perspective and enhances the transferability of findings to similar groups.

While the number of participants aligns with other lymphoedema research (Cooper‐Stanton et al. 2022), men remain underrepresented in health research compared to women (Markanday et al. 2013; Pathak et al. 2017). Future studies should investigate the experiences of Black, South, and East Asian men, as well as LGBTQ+ individuals, to ensure broader representativeness. Caution is warranted when transferring findings to contexts outside the UK and the Republic of Ireland, given differences in healthcare provision and cultural expectations of masculinity. The sample's demographic homogeneity, mostly White and heterosexual, limits applicability to more diverse populations. Nonetheless, our study offers important insights into the relational, physical, and psychological impacts of lymphoedema on men and the strategies they use to manage their condition.

## 5 Conclusion

This study explored men's daily experiences of living with non‐cancer‐related lymphoedema, revealing how the condition shaped identity, social relationships, and routine. Vulnerability and disruption were central themes, with participants describing the emotional and social consequences of stigma, misunderstanding, and loss of control. These challenges often disrupted their ability to perform traditionally masculine roles at work and in relationships.

Men responded in different ways. Some maintained traditional masculine traits such as stoicism and independence, while others redefined masculinity through acceptance, reflection, and relational support. By using theoretical frameworks such as hegemonic masculinity and the HIMM model, we gained insight into how men adapt and negotiate identity in the context of chronic illness. Future research should explore how other intersecting factors, such as ethnicity, sexuality, and class, influence men's experiences of lymphoedema. Further investigation may also be useful in other chronic conditions that affect identity, help‐seeking, and self‐management.

### 5.1 Relevance for Clinical Practice

This study highlights the importance of recognizing the specific challenges men face when living with lymphoedema. The condition disrupted routines, roles, and social participation—areas often tied to masculine identity. Participants developed structured routines to restore autonomy and manage symptoms, but many remained reluctant to seek support due to stigma and fear of misrecognition. Healthcare professionals should consider how gender norms influence how men experience illness, disclose symptoms, and engage with services. Clinical care must therefore be gender‐sensitive, inclusive, and responsive to men's psychosocial as well as physical needs. Building awareness, improving communication, and reducing stigma are essential steps in empowering men to manage lymphoedema effectively and maintain their well‐being.

## Author Contributions

Garry Cooper led the conceptualization, methodology, investigation, formal analysis, original draft writing, and review and editing of the manuscript. Nicola Gale contributed to the conceptualization, methodology, analysis, and review and editing. Manbinder Sidhu contributed to the conceptualization, methodology, analysis, and review and editing. Kerry Allen contributed to the conceptualization, methodology, analysis, and review and editing.

## Ethics Statement

The University of Birmingham Ethics Committee granted ethical approval for this (Decision number and date: ERN_20–1729/August 18, 2021). We assigned pseudonyms to all participants and coded their data to ensure anonymity (see Table 1). The study adhered to the ethical principles outlined in the Declaration of Helsinki (World Medical Association 2024). We managed data in accordance with the Data Protection Act and the EU General Data Protection Regulation, storing all materials on password‐protected computers (UK Government 2018). Participants received no financial incentives for their involvement.

## Consent

All participants read the participant information sheet and consented to the study. All data was coded for anonymity purposes with the removal of identifiable features and the use of pseudonyms.

## Conflicts of Interest

The authors declare no conflicts of interest.

## Data Availability

The data that support the findings of this study are available on request from the corresponding author. The data are not publicly available due to privacy or ethical restrictions.
